# Intra-articular Hyaluronic Acid Injections May Be Beneficial in Patients with Less Advanced Knee Osteoarthritis: A Systematic Review of Randomised Controlled Trials

**DOI:** 10.1007/s40279-025-02265-8

**Published:** 2025-07-02

**Authors:** Filippo Migliorini, Nicola Maffulli, Francesco Simeone, Naveen Jeyaraman, Swaminathan Ramasubramanian, Madhan Jeyaraman

**Affiliations:** 1 Department of Trauma and Reconstructive Surgery, University Hospital of Halle, Martin-Luther University Halle-Wittenberg, Ernst-Grube-Street 40, 06097 Halle (Saale), Germany; 2 Department of Orthopaedic and Trauma Surgery, Academic Hospital of Bolzano (SABES-ASDAA), Via Lorenz Böhler 5, 39100 Bolzano, Italy; 3 Department of Life Sciences, Health, and Health Professions, Link Campus University, Via del Casale di San Pio V, 00165 Rome, Italy; 4 Department of Trauma and Orthopaedic Surgery, Faculty of Medicine and Psychology, University La Sapienza, 00185 Rome, Italy; 5 School of Pharmacy and Bioengineering, Keele University Faculty of Medicine, Stoke on Trent, ST4 7QB UK; 6 Centre for Sports and Exercise Medicine, Barts and the London School of Medicine and Dentistry, Mile End Hospital, Queen Mary University of London, London, E1 4DG UK; 7 Department of Orthopaedics, ACS Medical College and Hospital, Dr MGR Educational and Research Institute, Chennai, Tamil Nadu 600077 India; 8 Department of Orthopaedics, Government Medical College, Omandurar Government Estate, Chennai, Tamil Nadu 600002 India

## Abstract

**Background:**

Knee osteoarthritis is a degenerative joint disease that impairs quality of life. Hyaluronic acid (HA) injections are used to restore synovial fluid viscosity and improve joint function.

**Objectives:**

The present systematic review investigated the prognostic factors influencing the effect of intra-articular HA injections for knee osteoarthritis (OA). The endpoint of interest was whether patient characteristics and molecular weight of the HA influence patient-reported outcome measures (PROMs) at different follow-ups.

**Methods:**

This study was conducted according to the Preferred Reporting Items for Systematic Reviews and Meta-Analyses: the 2020 PRISMA statement. All randomised controlled trials (RCTs) investigating the efficacy of intra-articular HA injections in the knee were accessed. Data concerning the visual analogue scale (VAS), Western Ontario and McMaster Universities Arthritis Index (WOMAC) and Lequesne scales were collected at baseline and the last follow-up. OA was scored using the Kellgren–Lawrence (KL) classification. The endpoint of interest was whether patient characteristics and the molecular weight of HA influence clinical outcomes.

**Results:**

The study included 71 RCTs and data from 10,590 patients; 67% (7082 of 10,570) were women. The mean age of the patients was 61.8 ± 5.1 years, and the mean body mass index (BMI) was 27.8 ± 2.3 kg/m^2^.

**Conclusions:**

HA injections lead to an initial worsening of symptoms; however, patients with early stage osteoarthritis, particularly older women, may experience significant long-term improvements. Further research should standardise treatment protocols and investigate the role of HA molecular weight in optimising outcomes.

**Supplementary Information:**

The online version contains supplementary material available at 10.1007/s40279-025-02265-8.

## Key Points

**Table:** 

The impact of molecular weight, patient demographics, disease severity and the use of different patient-reported outcome measures in patients who undergo intra-articular injections of hyaluronic acid (HA) for knee osteoarthritis is debated.
During the first 4 weeks following HA injections, patients with early-stage osteoarthritis eventually show improvement, whereas patients with advanced osteoarthritis report worsening patient‐reported outcome measures.
Patients who undergo intra-articular injections of hyaluronic acid in the knee with advanced osteoarthritis reported worsening patient-reported outcome measures.
Older women who undergo intra-articular injections of hyaluronic acid for knee osteoarthritis reported favourable outcomes in pain and patient-reported outcome measures.

## Introduction

Knee osteoarthritis (OA) is a pervasive degenerative joint disease that primarily affects the articular cartilage, leading to significant pain, stiffness, and functional limitations [1–4]. This condition is becoming increasingly prevalent with the ageing population, representing a significant cause of disability worldwide [2, 5–8]. Among various therapeutic interventions, intra-articular hyaluronic acid (HA) injections have been proposed to alleviate symptoms by enhancing joint lubrication and modulating the local inflammatory responses within the synovial environment [9, 10]. OA affects the entire joint, including significant alterations in synovial fluid composition, which HA injections aim to restore. Despite their widespread clinical use, the effectiveness of HA injections has been the subject of debate, reflecting a substantial heterogeneity in outcomes across numerous studies [11, 12]. The primary mechanism by which HA injections function involves supplementing the viscous properties of synovial fluid, thereby facilitating smoother joint movements and potentially slowing the degenerative process [13]. However, systematic reviews and randomised controlled trials (RCTs) have provided inconclusive results regarding the efficacy of HA injections, particularly concerning pain relief and functional improvement [14–17]. These discrepancies may be attributed to various factors, including the molecular weight of HA preparations, the stages of OA in the study populations and the methodological differences in trial design.

The molecular weight of HA could play a critical role in its therapeutic efficacy [18–21]. HA products are categorised on the basis of their molecular weight into low, medium and high molecular weight preparations, each purported to have different biological effects and side effect profiles [22, 23]. Hyaluronic acid formulations are categorised as low (approximately 500–730 kDa), medium (800–2000 kDa) and high (> 2000 kDa) molecular weights [24]. For instance, high molecular weight compounds are hypothesised to have longer intra-articular residence times and potentially more significant symptomatic relief, albeit at the increased risk of local adverse reactions [25, 26]. However, some studies have reported no significant differences in pain alleviation between high- and lower-molecular-weight HA products over extended periods, raising questions about the clinical relevance of using higher molecular-weight HA. The effectiveness of HA injections is also influenced by the outcome measures used to evaluate their impact [27, 28]. Commonly employed patient-reported outcome measures (PROMs), such as the Western Ontario and McMaster Universities Osteoarthritis Index (WOMAC) and the Lequesne scales, provide comprehensive assessments of pain, stiffness and physical function [29, 30]. These indices are pivotal for capturing the subjective experiences of patients undergoing HA treatments, and variations in these metrics could significantly influence the perceived effectiveness of the interventions [31]. Despite the theoretical benefits of HA injections, the literature reveals a considerable debate regarding their overall superiority compared with other conservative treatments such as physical therapy, exercise or even placebo [32–38]. The variability in response among patients underscores the necessity for personalised treatment approaches, which consider individual patient characteristics and disease severity.

Considering the current landscape of evidence and the ongoing controversies surrounding intra-articular HA therapy for knee OA, there is a critical need for a robust systematic review. Such an analysis should focus on elucidating the factors that influence the efficacy of HA injections, including the impact of HA molecular weight, patient demographics, disease severity and the use of different PROMs. By systematically evaluating and synthesising data from existing studies, a synthesis of evidence could significantly contribute to narrowing the knowledge gaps in this area, ultimately guiding clinical practice by identifying the subgroups of patients most likely to benefit from HA therapy and informing decisions regarding the optimal HA preparations to use in various clinical scenarios. The present systematic review investigated the prognostic factors of intra-articular HA injections for knee OA. The endpoint of interest was whether patient characteristics and molecular weight of the HA influence clinical outcomes.

## Methods

### Eligibility Criteria

All of the randomised controlled trials (RCTs) investigating the efficacy of intra-articular HA injections in the knee were accessed. Only studies published in peer-reviewed journals were considered. According to the authors' language capabilities, English, German, Italian, French and Spanish articles were eligible. Only studies with level I evidence, according to the Oxford Centre of Evidence-Based Medicine [39], were considered. Studies which evaluated intra-articular HA injections augmented with other biologically active compounds were not considered. Studies which evaluated intra-articular HA injections combined with experimental protocols (e.g. surgical, pharmacological and physiotherapeutic) were not considered. Studies which did not clearly state that injection was given in the knee were not eligible. Studies which did not report quantitative data under the outcomes of interest were not considered.

### Search Strategy

This study was conducted according to the Preferred Reporting Items for Systematic Reviews and Meta-Analyses: the 2020 PRISMA statement [40]. The PICOD algorithm was preliminarily established:

- Problem (P): knee OA
- Intervention (I): intra-articular HA injections
- Comparison (C): patient demographics and infiltrative protocol
- Outcomes (O): visual analogue scale (VAS), WOMAC and Lequesne scales
- Design (D): RCT

In January 2025, PubMed, Web of Science and Embase databases were accessed. No time constraint was set for the search. The Medical Subject Headings (MeSH) used for the database search are reported in the Appendix. No additional filters were used in the database search.

### Selection and Data Collection

Two authors (F.M. and F.S.) performed the database search. All the resulting titles were screened by hand, and the abstract was accessed if suitable. The full text of the abstracts, which matched the topic of interest, was accessed. If the full text was not accessible or available, the article was not considered for inclusion. A cross-check of the bibliography of the full-text articles was also performed to identify any further studies. Disagreements were debated and mutually resolved by the authors. In cases of further disagreements, a third senior author (N.M.) made the final decision.

### Data Items

Two authors (F.M. and F.S.) performed data extraction. The following data at baseline were extracted: author, year of publication and journal, length of the follow-up, number of patients with related sex, mean age and BMI. For each group, data on the molecular weights of the HA were extracted. Data concerning the following PROMs were collected at baseline and the last follow-up: visual analogue scale (VAS) at rest and during exercise [41], overall WOMAC score and related subscales of pain, stiffness and function [27] and Lequesne scales [42]. Data were extracted in Microsoft Office Excel version 16.72 (Microsoft Corporation, Redmond, USA). Concerning the WOMAC score, 24 health-specific items covering pain (5 items), stiffness (2 items) and function (17 items) were assessed. The subscale scores for pain, stiffness and function were summed to produce the total score. Scores ranged from 0 (no pain) to 20 (highest pain) for pain, 0 (no stiffness) to 8 (no stiffness) for stiffness, 0 (best function) to 68 (worst function) for function and 0 (best health) to 96 (worst health) for the total score. For the Lequesne scales, an interview of 11 questions about pain, discomfort and function was used. The score ranges from 0 (no pain, no disability) to 24 (maximum pain and disability). OA was scored using the Kellgren–Lawrence (KL) classification, which uses five grades ranging from 0 to 4 according to severity [43]. Results from each RCT were grouped according to the following follow-ups: 2 weeks–1 month, 5 weeks–2 months, 12–16 weeks and 17 weeks–6 months. The endpoint of interest was whether patient characteristics (mean age, mean BMI, sex and degree of OA) and molecular weight (kDa) of the HA influence the PROMs (VAS, WOMAC and Lequesne) at different follow-ups (2 weeks–4 weeks, 5 weeks–12 weeks, 13–16 weeks and 17 weeks–6 months).

### Methodological Quality Assessment and Quality of the Recommendations

The risk of bias was evaluated following the guidelines in the Cochrane Handbook for Systematic Reviews of Interventions [44]. Two reviewers (F.M. and F.S.) assessed the risk of bias in the extracted studies. Disagreements were resolved by a third senior author (N.M.). RCTs were evaluated using the revised risk of bias assessment tool (RoB2) [45, 46] of the Cochrane tool for assessing the risk of bias in randomised trials (RoB). The following endpoints were evaluated: bias arising from the randomisation process, bias owing to the deviations from intended interventions, bias because of missing outcome data, bias in the measurement of the outcome and bias in the selection of the reported result.

### Synthesis Methods

The main author (F.M.) performed the statistical analyses following the recommendations of the Cochrane Handbook for Systematic Reviews of Interventions [47]. The IBM SPSS software version 25 (International Business Machines Corporation, Armonk, USA) was used for descriptive statistics. A multiple pairwise analysis was performed to assess associations between patient characteristics (mean age, mean BMI, sex and degree of OA), molecular weight (kDa) of the HA and the PROMs (VAS, WOMAC and Lequesne) at different follow-ups (2 weeks–1 month, 5 weeks–2 months, 12–16 weeks, and 17 weeks–6 months). Categorical variables (sex, KL) were expressed as percentages. The STATA Software/MP version 16 (StataCorporation, College Station, Texas, USA) was used. A multiple linear model regression analysis through the Pearson product–moment correlation coefficient ($r$) was used. The Cauchy–Schwarz formula was used for inequality: + 1 is a positive linear correlation and − 1 negative. Values of 0.1 <|$r$|< 0.3, 0.3 <|$r$|< 0.5 and |$r$|> 0.5 were considered weak, moderate and strong correlations, respectively. The overall significance was assessed through the *χ*^2^ test, with values of *P* < 0.05 considered statistically significant.

## Results

### Study Selection

**Fig. 1:**
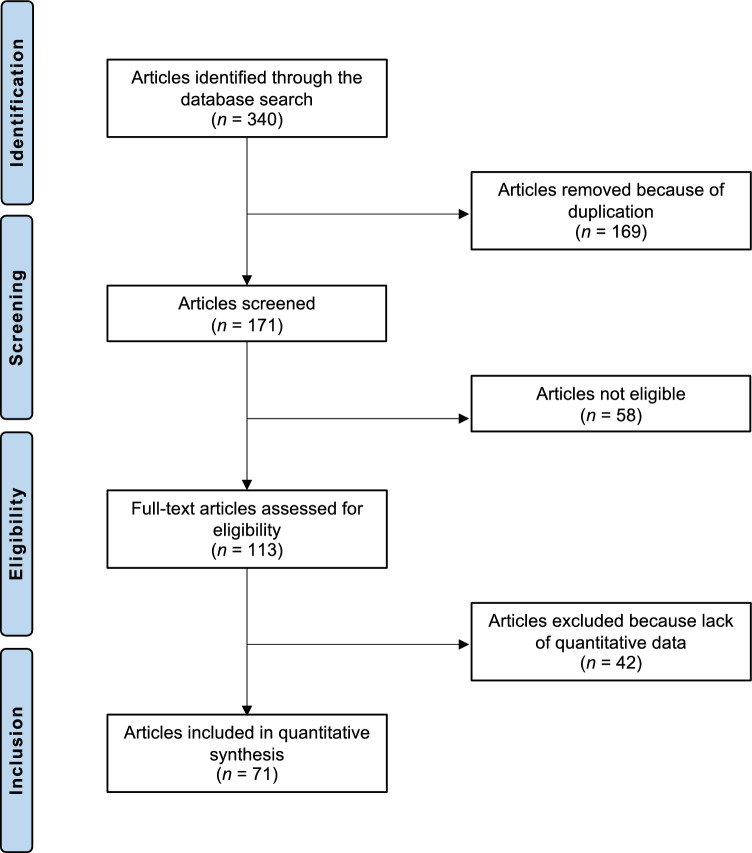
PRISMA flow chart of the literature search

The systematic literature search identified 340 clinical trials addressing the topic of interest. Of them, 169 studies were identified as duplicates and therefore excluded. The abstracts of the remaining 171 investigations were screened for eligibility. An additional 58 studies were discarded for lack of eligibility. In detail, the reasons for exclusion were inappropriate study type and design (*N* = 28), low level of evidence (*N* = 11), evaluating intra-articular HA injections combined with experimental protocols (*N* = 9), not clearly stating that injection was given in the knee (*N* = 6) and language limitations (*N* = 4). A further 42 studies did not include quantitative data on the endpoints of interest and were therefore not considered. This left 71 RCTs for final inclusion. The results of the literature search are shown in Fig. 1.

### Methodological Quality Assessment

**Fig. 2:**
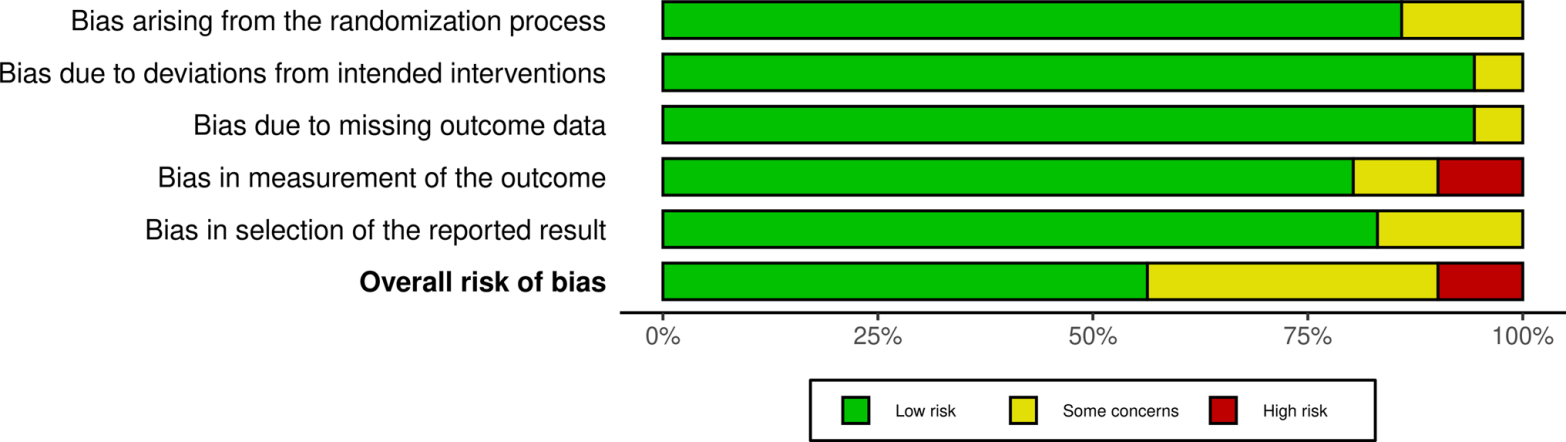
Cochrane risk of bias tool (RoB2) results

The revised Cochrane risk of bias assessment tool (RoB2) was utilised to investigate the risk of bias in all investigations included in the present review, since they were RCTs. The assessment identified some concerns during the randomisation process. However, given the established comparability of the groups studied at baseline, bias arising from the randomisation process was rated as predominantly low risk. Risk of bias based on the deviations from the intended intervention, missing outcome data, the selection of the reported outcome and the measurement of the outcome were occasionally noted with some concerns, leading to a low-to-moderate overall risk of bias in these domains. Given the lack of investigator blinding, several of the articles found a high risk of bias in outcome measurement; in all other studies, a low-to-medium risk was found for this area. In conclusion, the risk of bias graph evidenced a predominately good quality of the methodological assessment of the RCTs (Fig. 2).

### Study Characteristics and Results of Individual Studies

Data from 10,590 patients were collected; 67% (7,082 of 10,570) patients were women. The mean age of the patients was 61.8 ± 5.1 years, and the mean BMI was 27.8 ± 2.3 kg/m^2^. The generalities and demographics of the included studies are shown in Table 1.

**Table 1:** Generalities and demographics of the included studies

Study	Journal	Intervention	Patients (*n*)	Mean age	Women (%)	Mean BMI (kg/m^2^)
Altman et al., 2004 [48]	*Osteoarthritis Cartilage*	UHMW	173	62.9	46	
		Placebo	174	63.3	64	
Altman et al., 2009 [49]	*Semin Arthritis Rheum*	HMW	293	62.5	63	32.4
		Placebo	295	60.8	63	33.0
Arden et al., 2014 [50]	*Curr Med Res Opin*	UHMW	108	64.5	55	
		Placebo	110	60.9	46	
Arliani et al., 2021 [51]	*Rev Bras Ortop (Sao Paulo)*	PRP	14	62.8	79	28.3
		HMW	15	63.4	87	28.1
Bahrami et al., 2020 [52]	*BMC Musculoskelet Disord*	MMW	44	56.0	72	27.1
		LMW	46	59.5	75	27.5
Berenbaum et al., 2012 [25]	*Ann Rheum Dis*	LMW	217	67.2	14	28.0
		LMW	209	66.1	13	27.7
Bongkotphet et al., 2009 [53]	*Journal of Health Research*	MMW	32	64.8	69	26.1
		HMW	32	64.8	69	26.1
Buendía‐López et al., 2019 [54]	*J Orthop Traumatol*	PRP	35	56.2	52	24.9
		UHMW	36	56.6	53	24.9
		NSAIDs	35	57.4	52	25.2
Cerza et al., 2012 [55]	*Am J Sports Med*	PRP	60	66.5	58	
		LMW	60	66.2	53	
Chen et al., 2021 [56]	*Stem Cell Res Ther*	HMW	8	70.5	63	25.5
		MSCs	17	67.7	82	27.7
		MSCs	17	68.6	88	26.7
		MSCs	15	64.9	80	25.7
Cole et al., 2016 [57]	*Am J Sports Med*	PRP	52	55.9	43	27.4
		HMW	59	56.8	60	29.0
Cubukçu et al., 2005 [58]	*Clin Rheumatol*	HMW	20	52.6	70	
		Placebo	10	57.6	100	
De Campos et al., 2013 [59]	*Clin Orthop Relat Res*	HMW	52	61.0	75	30.0
		HMW and CCs	52	65.0	77	29.0
DeCaria et al., 2011 [60]	*Arch Gerontol Geriatr*	LMW	15	71.9	47	30.5
		Placebo	15	72.9	47	29.4
Diraçoğlu et al., 2009 [61]	*J Back Musculoskelet Rehabil*	HMW	42	59.4	90	31.1
		Placebo	21	56.2	100	31.3
Diraçoğlu et al., 2016 [62]	*J Back Musculoskelet Rehabil*	MMW	21	58.0	80	30.5
		LMW	20	56.4	85	30.8
Dougados et al., 1993 [63]	*Osteoarthritis Cartilage*	LMW	55	67.0	78	
		Placebo	55	69.0	65	
Dulic et al., 2021 [64]	*Medicina (Kaunas)*	MSCs	123	56.9	49	28.6
		HMW	35	59.4	57	30.0
		PRP	37	58.8	29	28.5
Duymus et al., 2017 [65]	*Knee Surg Sports Traumatol Arthrosc*	PRP	41	60.4	97	27.6
		MMW	40	60.3	97	28.4
		Ozone	39	59.4	89	27.6
Galluccio et al., 2021 [19]	*Ther Adv Musculoskelet Dis*	LMW	30	66.0	47	
		LMW	30	64.0	47	
		LMW	30	64.0	57	
Gigis et al., 2016 [66]	*Hippokratia*	HMW	40	67.2	58	
		LMW	40	67.4	63	
Guler et al., 2015 [12]	*Eur J Orthop Surg Traumatol*	MMW	86	55.1	89	28.6
		PRP	89	55.0	80	28.4
Guo et al., 2018 [67]	*Med Sci Monit*	UHMW	129	64.8	77	27.4
		HMW	129	62.0	73	27.6
Ha et al., 2017 [68]	*BMC Musculoskelet Disord*	MMW	141	62.4	81	24.8
		UHWM	146	62.0	78	25.1
Hangody et al., 2018 [69]	*Cartilage*	LMW	149	57.5	65	28.9
		MMW	150	59.2	66	28.4
		Placebo	69	58.0	74	29.1
Henderson et al., 1994 [70]	*Ann Rheum Dis*	LMW	10	63.9	50	
		LMW	25	72.1	80	
		Placebo	20	60.0	75	
		Placebo	26	67.0	69	
Ho et al., 2022 [71]	*J Orthop Translat*	MSCs	10	56.7	60	25.4
		HMW	10	59.1	80	26.0
Huang et al., 2011 [72]	*BMC Musculoskelet Disord*	LMW	100	65.9	74	25.7
		Placebo	100	64.2	78	25.4
Huang et al., 2021 [73]	*BMC Musculoskelet Disord*	UHMW	71	56.6	65	
		LMW	71	56.0	71	
Huang et al., 2019 [74]	*Orthopade*	LMW	40	54.8	84	24.5
		LMW and CCs	40	54.3	83	24.6
		LMW and PRP	40	54.5	79	25.2
Huskisson et al., 1999 [75]	*Rheumatology (Oxford)*	LMW	50	65.8	76	
		Placebo	50	64.8	58	
Jüni et al., 2007 [76]	*Arthritis Rheum*	HMW	222	63.3	65	28.2
		MMW	219	63.5	69	28.1
		MMW	219	63.3	65	28.6
Karlsson et al., 2002 [77]	*Rheumatology (Oxford)*	LMW	92	72.0	67	
		HMW	88	70.0	65	
		Placebo	66	71.0	61	
Ke et al., 2021 [78]	*BMC Musculoskelet Disord*	Placebo	220	61.6	78	25.4
		HMW	218	61.5	77	25.6
Khanasuk et al., 2012 [79]	*J Med Assoc Thai*	HMW	16	65.1	80	26.6
		LMW	16	67.0	80	25.4
Kim et al., 2023 [80]	*Sci Rep*	IA PN	30	63.6	83	
		MMW	30	65.4	75	
Ko et al., 2022 [81]	*Pharmaceutics*	UHMW	71	66.1	79	
		UHMW	71	65.5	82	
Kraeutler et al., 2021 [82]	*Orthop J Sports Med*	LMW	16	53.6	23	23.5
		PRP	20	53.3	56	23.7
Lin et al., 2019 [83]	*Arthroscopy*	PRP	31	61.2	71	24.0
		MMW	27	62.5	66	26.6
		Placebo	29	62.2	63	25.0
Louis et al., 2018 [84]	*Arthroscopy*	PRP	24	53.2	42	25.6
		UHMW	24	48.5	54	27.0
Maheu et al., 2019 [85]	*PLoS One*	MMW	144	67.1	72	26.4
		HMW	148	66.6	61	26.3
Maia et al., 2019 [86]	*Clinics (Sao Paulo)*	MMW	16	56.6	63	31.9
		MMW and CCs	16	54.5	63	29.0
		CCs	12	60.3	92	31.4
Martin et al., 2016 [87]	*BMC Musculoskelet Disord*	Collagen	32	69.4	86	27.2
		LMW	32	70.0	65	27.3
Mochizuki et al., 2020 [88]	*Asia Pac J Sports Med Arthrosc Rehabil Technol*	LMW	37	69.0	68	23.1
		MMW	36	65.2	71	24.5
Moon et al., 2023 [89]	*Pain Med*	LMW	30	67.5	63	
		MMW	30	67.5	70	
		UHMW	30	67.0	93	
Ozcamdalli et al., 2017 [90]	*Cartilage*	HMW	10			
		NAC	10			
Park et al., 2021 [91]	*Am J Sports Med*	PRP	55	60.6	71	25.5
		UHMW	55	62.3	85	25.9
Paterson et al., 2016 [92]	*BMC Musculoskelet Disord*	PRP	12	49.9	27	27.9
		HMW	11	52.7	30	30.9
Petrella et al., 2002 [93]	*Arch Intern Med*	LMW	30	67.3	36	29.5
		LMW and NSAIDs	30	65.0	45	31.6
		NSAIDs	30	66.3	42	29.4
		Placebo	30	62.6	43	32.7
Petrella et al., 2015 [94]	*BMC Musculoskelet Disord*	Hydros	33	59.0	63	29.8
		HMW and CCs	33	61.0	59	29.0
		HMW	32	59.0	50	29.0
Petterson et al., 2019 [95]	*Knee Surg Sports Traumatol Arthrosc*	MMW	184	59.5	59	29.9
		Placebo	185	58.7	57	30.4
Pham et al., 2004 [96]	*Ann Rheum Dis*	MMW	131	64.9	71	
		Diacerin	85	64.5	69	
		Placebo	85	64.9	61	
Raeissadat et al., 2015 [97]	*Clin Med Insights Arthritis Musculoskelet Disord*	PRP	87	56.9	10	28.2
		LMW	73	61.1	24	27.0
Raeissadat et al., 2017 [98]	*Clin Med Insights Arthritis Musculoskelet Disord*	PRGF	41	57.0	82	28.6
		LMW	36	59.5	82	27.5
Raeissadat et al., 2018 [99]	*J Pain Res*	Ozone	87	58.1	75	26.8
		LMW	87	61.1	76	28.6
Raeissadat et al., 2020 [100]	*J Pain Res*	PRGF	60	57.1	72	27.9
		LMW	59	58.6	71	28.7
Raeissadat et al., 2021 [101]	*BMC Musculoskelet Disord*	PRP	59	56.1	73	27.5
		PRGF	60	57.9	76	27.5
		LMW	59	56.1	75	27.4
		Ozone	60	57.6	75	27.0
Sanchez et al., 2012 [102]	*Arthroscopy*	PRGF	89	60.5	52	27.9
		HMW	87	58.9	52	28.2
Sconza et al., 2023 [103]	*Int J Mol Sci*	Ozone	26	68.0	42	29.1
		LMW	26	68.0	62	27.9
Shimizu et al., 2010 [104]	*J Orthop Sci*	LMW	32	75.9	78	
		CCs	29	75.3	73	
Su et al., 2018 [105]	*Clin Rheumatol*	PRP	28	50.7	63	28.2
		PRP	26	54.2	56	28.2
		LMW	32	53.1	60	28.7
Sun et al., 2017 [106]	*J Bone Joint Surg Am*	UHMW	66	62.7	77	24.7
		HMW	66	62.5	71	25.2
Tammachote et al., 2016 [107]	*J Bone Joint Surg Am*	HMW	55	62.6	86	26.3
		CCs	55	61.0	73	25.8
Tasciotaoglu et al., 2003 [108]	*Clin Rheumatol*	MMW	30	57.4		32.7
		CCs	30	60.1		33.3
Vanelli et al., 2010 [109]	*Knee Surg Sports Traumatol Arthrosc*	Polynucleotides	30	60.0	66	26.7
		LMW	30	67.0	67	28.8
Waluyo et al., 2021 [110]	*J Rehabil Med*	Prolotherapy	44	62.6	77	
		LMW	32	62.0	71	
Wang et al., 2018 [111]	*Exp Ther Med*	CCs	60	63.6	77	25.3
		LMW	60	62.5	73	26.0
Wang et al., 2022 [112]	*Medicina (Kaunas)*	PRP	58	61.9	78	24.1
		UHMW	58	63.0	71	24.0
van der Weegen et al., 2015 [17]	*J Arthroplasty*	MMW	99	58.7	51	28.6
		Placebo	97	60.1	48	29.3
Yaradilmis et al., 2020 [113]	*J Orthop*	MMW	35	63.0	87	32.4
		PRP	36	60.3	87	31.3
		PRP	34	58.9	90	32.5
Zhang et al., 2015 [114]	*Arthritis Res Ther*	LMW	174	60.4	80	
		UHMW	175	60.2	74	

*CCs* corticosteroids, *PRP* platelet-rich plasma, *MSC* mesenchymal stem cell, *UHMW* ultra-high molecular weight, *HMW* high molecular weight, *MMW* medium molecular weight, LMW low molecular weight, *PRGF* platelet-rich in growth factors, *NSAIDs* nonsteroidal anti-inflammatory drugs, *FU* follow-up

### Results Syntheses

Age demonstrated a significant negative association with pain and functional outcomes across multiple timepoints. Specifically, age was inversely associated with VAS scores (*r* = − 0.7) and WOMAC total scores (*r* = − 0.8) between 5 and 8 weeks post-intervention. This negative association persisted at later follow-ups (17 weeks–6 months), where age was negatively correlated with VAS during exercise (*r* = − 0.7), WOMAC total (*r* = − 0.4) and WOMAC-function subscale scores (*r* = − 0.5). Female sex was also significantly negatively associated with several WOMAC subscales. At 5–8 weeks, female patients exhibited lower WOMAC-pain (*r* = − 0.8), WOMAC-stiffness (*r* = − 0.9) and WOMAC-function (*r* = − 0.8) scores. These negative associations remained significant at 12–16 weeks for WOMAC total (*r* = − 0.5), WOMAC-pain (*r* = − 0.7), WOMAC-stiffness (*r* = − 0.9) and WOMAC-function (*r* = − 0.6). Similarly, from 17 weeks to 6 months, female sex continued to be negatively associated with WOMAC-pain (*r* = − 0.7), WOMAC-stiffness (*r* = − 0.7) and WOMAC-function (*r* = − 0.7). As classified by the KL grading system, radiographic severity of OA showed distinct associations with clinical outcomes. KL grade I was strongly positively associated with Lequesne index scores at 2–4 weeks (*r* = 0.9). KL grade II demonstrated a positive association with VAS scores at 5–8 weeks (*r* = 0.9) but was negatively associated with WOMAC-stiffness at 12–16 weeks (*r* = − 0.6) and with WOMAC-pain (*r* = − 0.4) and WOMAC-stiffness (*r* = − 0.5) from 17 weeks to 6 months. KL grade III was negatively associated with WOMAC-stiffness at 1–16 weeks (*r* = − 0.6) and 17 weeks to 6 months (*r* = − 0.4). KL grade IV correlated positively with VAS (*r* = 0.7) and Lequesne index (*r* = 0.9) at 2–4 weeks and with VAS during exercise at both 12–16 weeks and 17 weeks–6 months (*r* = 0.5 for both). The molecular weight (kDa) was negatively associated with WOMAC-stiffness scores at 2–4 weeks (*r* = − 0.7) and with VAS scores at 12–16 weeks (*r* = − 0.4). No additional statistically significant associations were found. An overview of the pairwise correlations is reported in Table 2.

**Table 2:** Results of the pairwise analyses (*: < 0.05 |*P*|> 0.005; **: < 0.005 |*P*|> 0.0001; ***: *P* < 0.0001)

Endpoints	Age (years)	Women (%)	BMI (kg/m^2^)	KL-I (%)	KL-II (%)	KL-III (%)	KL-IV (%)	kDa
**2–4 weeks**								
VAS at rest	− 0.2	− 0.1	− 0.4	0.1				0.1
VAS at exercise	− 0.3	0.4	0.0	− 0.4				0.0
VAS	0.1	0.2	− 0.2	0.1	− 0.6	0.4	0.7**	− 0.3
WOMAC	− 0.1	− 0.4	0.0	− 0.1	− 0.1	− 0.2	0.3	− 0.2
WOMAC-pain	− 0.4	0.3	− 0.2	− 0.3	0.1	0.4		0.0
WOMAC-stiffness	− 0.5	− 0.1		− 0.2	0.2	0.0		− 0.7**
WOMAC-function	− 0.5	0.1	− 0.2	− 0.4	0.6*	0.4		− 0.1
Lequesne	− 0.1	− 0.1	0.3	0.9**	− 0.7	− 0.3	0.9**	− 0.4
**5–12 weeks**								
VAS at rest	0.1	0.8						0.4
VAS at exercise	0	0.7				0.9	0.7	0.5
VAS	− 0.7*	0.2	0.5	0.1	0.9**	− 0.4	− 0.7	− 0.2
WOMAC	− 0.8**	− 0.6	0.1	− 0.2	− 0.8	0.1	− 0.3	− 0.4
WOMAC-pain	− 0.4	− 0.8*	− 0.7		− 0.8	0.4		− 0.1
WOMAC-stiffness	− 0.5	− 0.9*	− 0.8		− 0.8	0.4		− 0.1
WOMAC-function	− 0.6	− 0.8*	− 0.6		− 0.7	0.2		− 0.2
**13–16 weeks**								
VAS at exercise	− 0.5	0.9	0.3	− 0.0				0.1
VAS	− 0.1	− 0.3	0.2	0	− 0.4	0.2	0.5*	− 0.4**
WOMAC	− 0.4	− 0.5**	0.2	− 0.2	− 0.3	0.1	0.0	− 0.3
WOMAC-pain	− 0.3	− 0.7***	− 0.4	− 0.1	− 0.4	0.5	− 0.1	− 0.2
WOMAC-stiffness	− 0.3	− 0.7**	− 0.4	− 0.1	− 0.6*	0.6*		− 0.3
WOMAC-function	− 0.4	− 0.6**	− 0.1	− 0.3	− 0.4	0.4		− 0.4
Lequesne	0.4	− 0.5	0.2	0.6	− 0.5	− 0.2	0.6	− 0.5
**17 weeks–6 months**								
VAS at rest	0.4	0.2	0.0	− 0.2				0.5
VAS at exercise	− 0.7**	0.1	0.7	0.1	0.2	− 0.2		0.4
VAS	− 0.3	− 0.1	0.3	0.1	0.1	− 0.3	0.5**	− 0.1
WOMAC	− 0.4**	− 0.5	0.2	− 0.1	− 0.3	0.1	0.0	− 0.3
WOMAC-pain	− 0.4	− 0.7***	− 0.3	− 0.1	− 0.4*	0.4	0.0	− 0.2
WOMAC-stiffness	− 0.4	− 0.7***	− 0.3	− 0.1	− 0.5*	0.4*		− 0.2
WOMAC-function	− 0.5*	− 0.6**	− 0.1	− 0.2	− 0.4	0.2		− 0.3
Lequesne	0.3	0.5	0.3	0.3	− 0.4	0.2	0.3	− 0.4

*VAS* visual analogue scale, *WOMAC* Western Ontario and McMaster Universities Osteoarthritis Index, *BMI* body mass index, *KL* Kellgren–Lawrence classification, *kDa* molecular weight in kilodalton

## Discussion

The present systematic review demonstrated that during the first 4 weeks following HA injections, patients with advanced osteoarthritis experienced worsening patient‐reported outcome measures, while patients with early-stage osteoarthritis showed subsequent improvement. Additionally, subgroup analyses indicated that older women exhibited more favourable outcomes.

The aggregated analysis of data from 10,590 patients, predominantly females (67%), across various studies investigating intra-articular injections of HA for knee OA offers a rich dataset to explore demographic influences on treatment efficacy, intervention outcomes and longitudinal effects. This extensive cohort, with a mean age of 61.8 years and a mean BMI of 27.8 kg/m^2^, provides a sound basis to examine the interplay between age, sex, BMI and therapeutic outcomes. All patients in the present systematic review received HA injections for knee osteoarthritis.

Although the present investigation included variables such as patient sex and BMI, their role was secondary to the primary effect of HA injections on osteoarthritis outcomes. Discussion of these factors is limited to their potential impact on patient-reported outcomes. Intervention protocols varied across studies, including differences in HA molecular weight, injection frequency (single versus multiple injections) and administration techniques. The analysis revealed sex-specific responses, with female patients, particularly older women, exhibiting more favourable outcomes than male patients. Long-term follow-up was defined as assessments at 5–8 weeks and beyond. Initial improvements were most consistently observed in patients receiving high-molecular-weight HA compared with low-molecular-weight formulations. The high prevalence of osteoarthritis among the female participants, particularly post-menopausal, aligns with prior findings that suggest hormonal changes significantly influence joint health [115, 116]. This demographic trend is essential for understanding the disease pathophysiology and tailoring treatment approaches. High BMI, in the overweight range, is another critical factor, as it not only exacerbates mechanical stress on weight-bearing joints but also involves metabolic factors that may influence the progression of OA [117].

Various interventions have been used across the studies in the present investigation, including different molecular weights of HA, which are supposed to offer differing biomechanical and pharmacokinetic profiles. The larger molecular weight viscosupplements, such as ultra-high molecular weight (UHMW) and high molecular weight (HMW), are suggested to provide more robust joint lubrication and mechanical support, which could be particularly beneficial in joints with less severe synovial inflammation [48, 49]. In contrast, low molecular weight (LMW) and medium molecular weight (MMW) products might be preferable in highly inflamed joints given their enhanced penetrative abilities and possibly lower viscosities, facilitating better distribution within the synovial fluid [53, 118]. Regenerative treatments such as platelet-rich plasma (PRP) and mesenchymal stem cells (MSCs) aim to address the underlying pathologies of osteoarthritis, including cartilage degradation and subchondral bone remodelling [37, 119]. The efficacy of PRP varies, which could be related to differences in preparation methods and the concentration of growth factors [51]. MSCs have shown promise in modulating the joint environment and promoting cartilage regrowth [56]. The variability in WOMAC and VAS scores across studies highlights the influence of individual patient factors and disease severity on the outcomes of these interventions.

The pairwise correlations presented in the studies (Table 2) provide a nuanced view of how demographic factors such as age, sex and BMI impact the response to treatment. Notably, negative correlations between WOMAC-function scores and age suggest that younger patients experience better functional outcomes, likely from less advanced osteoarthritic changes and a higher intrinsic capacity for cartilage regeneration [60]. Meanwhile, correlations between the sex of the patients and VAS scores during exercise might reflect sex-specific responses to pain management, potentially influenced by hormonal factors or differences in pain perception [58]. Moreover, higher BMI is often correlated with poorer responses to physical interventions, highlighting the mechanical and possibly metabolic challenges in treating overweight and obese patients [25]. This insight is crucial to developing personalised treatment plans considering the patient’s mechanical load and systemic health.

Long-term follow-up results reveal significant insights into the durability of treatment effects. For instance, viscosupplementation shows initial improvements in WOMAC and VAS scores, which may not always be sustained, indicating the potential need for repeated treatments or combination therapies [66]. This observation underscores the importance of ongoing patient management and possibly step-up therapy strategies to maintain joint health and function. The findings also suggest that specific treatments may be better suited for certain stages of osteoarthritis, guided by the Kellgren–Lawrence (KL) classifications. Patients with advanced stages might respond differently to HA treatment compared with those in the earlier stages, potentially from differing structural damage and inflammation levels [76, 107].

Results from the present investigation indicate that increased HA molecular weight may be associated with improved outcomes; however, this effect appears more pronounced in older women than in men. Note that sex-specific mean ages were not uniformly reported, and these conclusions are made on the basis of available subgroup analyses. Patient age and female sex were inversely associated with PROMs from the 5-week to the 6-month follow-ups. There was no evidence of an association between BMI and PROMs. During the first 2–4 weeks of follow-up, there is evidence of a positive association between PROMs and all KL stages. Following the first 2–4 weeks of follow-up, there is evidence of a negative association between PROMs and the first two KL stages and a negative association with the last two KL stages. Limited evidence supports the association between increased molecular weight of HA and improved clinical outcomes. Taken together, the findings suggest older women might have greater benefits from injection therapy using HA. During the first month, irrespective of the progression of knee OA, patients might experience worsening outcomes. After the first month, patients with early-to-moderate OA might experience improved symptoms and function, and patients with advanced-to-severe OA might report worsening clinical outcomes. We hypothesised that the progression of joint degeneration in patients with advanced-to-severe OA might explain the worsening of symptoms and function. The influence of the molecular weights of HA on clinical outcomes remains unclear. The implications of these findings for clinical practice are profound. The demographic trends observed necessitate a sex-specific analysis of treatment outcomes, as hormonal variations could influence disease progression and the response to therapy. The association between demographics and treatment efficacy highlights the need for personalised medicine approaches in managing osteoarthritis, where each patient’s specific characteristics and needs are considered when designing therapeutic regimens.

The study of varying molecular weights of viscosupplements and biologics such as PRP and MSCs in the management of osteoarthritis underscores the evolving landscape of osteoarthritic treatments and the potential for innovative therapies to address the complex pathophysiology of the condition. The nuanced relationships between patient characteristics and treatment responses also highlight the importance of a more stratified approach to treatment, considering factors such as age, sex, BMI and the specific molecular properties of therapeutic agents.

Given the high heterogeneity, there is variability in study designs, follow-up durations and outcome measures, potentially impacting data comparability and pooling. A few studies did not score the severity of OA using the Kellgren–Lawrence classification system; consequently, they have not been considered in the evaluation of severity. Blinding participants and personnel in certain studies may introduce performance and detection biases, particularly for subjective outcomes such as pain and function. The restriction of eligible studies to languages understandable by researchers may omit relevant literature published in other languages, while publication bias may skew results towards more favourable outcomes. Additionally, using PROMs such as the WOMAC and VAS introduces subjective elements susceptible to patient perception and reporting biases, potentially misrepresenting treatment efficacy. Limited long-term follow-up in many studies leaves uncertainties regarding the sustained efficacy and safety of intra-articular HA injections beyond 6 months. Variations in dose regimens and injection techniques, including the number of injections and intervals between treatments, were noted across studies and may have contributed to differences in outcomes. Different dose and administration techniques across studies complicate result consistency, lacking standardised protocols. Moreover, the absence of direct comparisons with established therapies for knee osteoarthritis limits the understanding of the relative efficacy of HA injections, necessitating comparative effectiveness research.

## Conclusions

Patients should be advised of findings suggesting a temporary PROM worsening during the first 4 weeks following HA injections. However, PROM improvements are noted after this period in patients with early stage osteoarthritis; the effect of HA injections in those with advanced knee OA is more debatable. Notably, older women demonstrate favourable results in VAS and WOMAC scores. Despite the symptomatic relief provided by HA injections, the diversity in study designs and the lack of standardised treatment protocols significantly hinder the interpretation of these findings. Furthermore, the predominance of studies with short-term follow-up contributes to the limitations in assessing long-term efficacy. Higher molecular weight HA may be more beneficial for specific patient groups, yet evidence remains sparse. To overcome these limitations, future research should focus on long-term outcomes employing rigorous study designs and include comparative analyses with other treatment modalities. This approach will enable a more precise delineation of HA injections’ role in the comprehensive management of osteoarthritis and support the development of personalised, effective treatment protocols that cater to the specific needs of individual patients.

## Supplementary Information

Below is the link to the electronic supplementary material.

Supplementary file1 (DOCX 18 KB)

## Declarations

### Availability of data and materials

The datasets generated during and/or analysed during the current study are available throughout the manuscript.
