# Supplementary material for: Intra-articular Hyaluronic Acid Injections May Be Beneficial in Patients with Less Advanced Knee Osteoarthritis: A Systematic Review of Randomised Controlled Trials
Source: Sports Med. 2025 Jul 2;55(8):1953–69. doi: 10.1007/s40279-025-02265-8 (PMC12460378; doi:10.1007/s40279-025-02265-8)
Supplement: Supplementary file 1 — Supplementary file1 (DOCX 18 KB) [file 40279_2025_2265_MOESM1_ESM.docx]

**Systematic literature search for:**

**Predictors of the efficacy of intra-articular hyaluronic acid infiltrations in patients with knee osteoarthritis: a systematic review of RCTs**

**Concept 1: Knee osteoarthritis**

**Keywords:**

knee OA

knee osteoarthritis

**Mesh:**

"Osteoarthritis, Knee"[Mesh]

**Concept 2: Treatment**

**Keywords:**

hyaluronic acid infiltrations

HA infiltrations

**Mesh:**

"Hyaluronic Acid"[Mesh]

**Concept 3: Interest of the outcome**

**PROMs**

**Keywords:**

Western Ontario and McMaster Universities Osteoarthritis (WOMAC)

Lequesne scales

**Mesh:**

"Patient Outcome Assessment"[Mesh]

"Patient Reported Outcome Measures"[Mesh]

**Concept 1:**

"Osteoarthritis, Knee"[Mesh] OR knee osteoarthritis OR knee OA

**AND**

**Concept 2:**

"Hyaluronic Acid"[Mesh] OR hyaluronic acid infiltrations OR HA infiltrations

**AND**

**Concept 3:**

"Patient Outcome Assessment"[Mesh] OR "Patient Reported Outcome Measures"[Mesh] OR PROM OR Western Ontario and McMaster Universities Osteoarthritis OR WOMAC OR Lequesne scales

**NOT**

Hip

**Summary of the Search**

((("Osteoarthritis, Knee"[Mesh] OR knee osteoarthritis OR knee OA) AND ("Hyaluronic Acid"[Mesh] OR hyaluronic acid infiltrations OR HA infiltrations)) AND ("Patient Outcome Assessment"[Mesh] OR "Patient Reported Outcome Measures"[Mesh] OR PROM OR Western Ontario and McMaster Universities Osteoarthritis OR WOMAC OR Lequesne scales)) NOT (Hip)
